# Sustainable Materials and Structures Used in Pavement Engineering

**DOI:** 10.3390/ma18010205

**Published:** 2025-01-06

**Authors:** Fucheng Guo, Augusto Cannone Falchetto, Bochao Zhou, Wentong Wang

**Affiliations:** 1 School of Civil Engineering, Lanzhou Jiaotong University, Lanzhou 730070, China; 2 Department of Civil Engineering, Aalto University, 02150 Espoo, Finland; augusto.cannone@unipd.it; 3 Department of Road & Railway Engineering, Beijing University of Technology, Beijing 100124, China; bczhou@bjut.edu.cn; 4 Faculty of Transportation, Shandong University of Science and Technology, Qingdao 266590, China; wangwentong@chd.edu.cn

Sustainable materials and structures have become widely used in asphalt pavements to mitigate the resource crisis and achieve carbon neutrality. These materials include, but are not limited to, warm-/cold-mix asphalt (WMA/CMA), reclaimed asphalt pavement (RAP), and waste materials used in asphalt mixtures. These materials and structures can help save nonrenewable resources and improve the road environment. However, there are still several knowledge gaps to overcome, for example, the compatibility between new and old materials and structures and the long-term performance and cost performance of new materials and structures. These limitations hinder the mega-scale application of sustainable materials and structures in asphalt pavement construction.

This Special Issue, titled “Sustainable Materials and Structures Used in Pavement Engineering”, presents an overview of the current research on sustainable materials and structures used in pavement engineering. For this Special Issue, we received over 20 submissions within one collection year, and 11 high-quality articles were published after careful reviews and revisions were conducted by reviewers and authors.

Among these publications, six focus on sustainable materials used in pavement engineering. A multi-component solid waste cementitious material (SWCM) was designed by Wu et al. [1] based on the response surface method. The synergistic reaction mechanism of the SWCM was analyzed using an X-ray diffractometer (XRD), Fourier transform infrared spectroscopy (FT-IR), and thermogravimetric analysis (TG). A shrinkage testing system was developed to evaluate the anti-cracking characteristics of stable macadam using multiple solid waste cementitious materials (SWCM-SM), and the strength growth law and frost resistance were analyzed. The results showed that SWCM-SM exhibited a slower hydration reaction and longer hydration duration, demonstrating the characteristics of low early strength and high later strength. Wang et al. [2] focused on construction technology and road performance using recycled construction waste materials in urban road sub-base construction. Through indoor tests such as sieving and unconfined compressive strength tests, relevant technical indicators were obtained and analyzed. Additionally, periodic core sampling, compaction tests, and rebound deflection tests were conducted on-site according to relevant standards in order to thoroughly investigate the specific effects of using construction waste in practice and to analyze and evaluate the actual feasibility of the materials for road use. Yu et al. [3] performed an innovative examination of blending behavior between virgin asphalt and aged asphalt incorporating a new bio-based warm-mix rejuvenator (BWR) utilizing atomic force microscopy (AFM). Through analyzing the variation in several micro-morphology parameters between virgin asphalt and aged asphalt (or recycled asphalt) after blending, an index of regenerative blending degree (RBD) was proposed to quantitatively evaluate their blending behavior and the effect of various blending temperatures and durations on the regenerative blending degree was investigated. He et al. [4] evaluated the conventional physical properties, rheological performance, and micro-morphology of aged asphalt incorporating a new bio-based warm-mix rejuvenator (BWR) and a commercial warm-mix rejuvenator (ZJ-WR). The regeneration mechanism of warm-mix rejuvenators in aged asphalt was analyzed by Fourier transform infrared spectroscopy (FTIR). The results showed that the new bio-based warm-mix rejuvenator had a better regeneration effect on the performance and micro-morphology of aged asphalt than the commercial warm-mix rejuvenator. Jiang et al. [5] comprehensively explored the influence of chemical components on the fatigue performance of hard asphalt. Rheological, time sweep, and linear amplitude sweep (LAS) tests were carried out to evaluate the fatigue properties of seven types of hard asphalt. The results showed that the complex modulus of asphalt binded increasingly rapidly with an increase in asphaltene and resins and that the colloidal structure was strengthened, which would increase the fatigue factor. Linear regression analysis showed that the fatigue life of hard asphalt showed a good correlation with the strain sensitivity. To investigate the rheological properties and aging resistance of SBR-modified bitumen binder (SB) modified with Sasobit/waste cooking oil (Sasobit/WCO) compounds, Zhao et al. [6] selected three additives, Sasobit, WCO, and Sasobit/WCO composite, to assess their effects, as well as the effect of the temperature at which a mixture was prepared, on the physical and rheological characteristics of SB. In addition, the effects of this innovative warm-mix addition on the performance grade (PG) and aging resistances of SB were evaluated. According to the results, Sasobit/WCO composites outperformed Sasobit and WCO in terms of preparation temperature, high- and low-temperature performance, and good resistance to thermal cracking.

Five papers focus on sustainable technologies and structures used in pavement engineering. In order to reduce the porosity of emulsified asphalt mixtures, an innovative forming process was proposed by Xiao et al. [7] to improve its performance, and its strength formation mechanism was explored. Three groups of emulsified asphalt mixtures were prepared by a conventional mixing process and a novel mixing process. A Marshall test, computed tomography (CT) scanning test, and workability test and analysis were performed. The results show that, compared with conventional methods, the innovative forming method can increase the bulk density of the mixture and reduce its porosity, thus improving its technical performance. With regard to the effects of fiber on fracture behavior, the fracture behavior of asphalt mixtures with various fiber types and contents was studied by Li et al. [8] using the indirect tensile asphalt cracking test (IDEAL-CT) in conjunction with digital image correlation (DIC) technology. The different evaluation indexes used in the test were obtained using digital image correlation technology. The optimum fiber contents were determined, specifically, 0.4%, 0.3%, and 0.3% for basalt fiber, glass fiber, and polyester fiber, respectively. A ultra-high performance concrete (UHPC) beam shear test database containing 247 samples was created by Gao et al. [9], and the influencing factors on the shear capacity of UHPC beams, such as the shear span ratio, the web reinforcement ratio, and the volume fraction of steel fiber, were analyzed. It was found that the ratio of the cracking load to the ultimate load ranged from 0.2 to 0.6. Moreover, a formula for calculating the shear-bearing capacity of UHPC beams with and without web reinforcement was proposed and verified with higher prediction accuracy. Song et al. [10] explored the distribution of surface macrotexture with depth in open-graded friction course (OGFC). Using cross-sectional images and semantic image segmentation techniques, the internal structure, porosity, and void size distribution were analyzed to assess the effectiveness of rainfall drainage. Skid resistance was evaluated with a British Pendulum Tester, focusing on the influence of the surface macrotexture and internal macrostructure, particularly with regard to contact depth. Jiao et al. [11] employed molecular dynamics (MD) methods to obtain quasi-liquid layer (QLL) thicknesses and utilized these measurements to estimate the adhesive strength between ice and asphalt. The QLL thickness was determined for various asphalts and temperatures using the tetrahedral order parameter gradient. The findings were then compared with ice adhesion strength data acquired from pull-off tests.

It is well known that more and more sustainable materials and structures will be used in pavement engineering against the background of the dual carbon strategy and sustainable development. While the papers and research in this Special Issue are highly insightful, we only collected limited research in this area. However, we believe that these papers offer a good overview of the sustainable materials and structures used in pavement engineering. We hope that further research will focus on this field, to contribute to worldwide progress with regard to sustainable development.
